# Supplementary material for: Clinical and Genetic Re-Evaluation of Inherited Retinal Degeneration Pedigrees following Initial Negative Findings on Panel-Based Next Generation Sequencing
Source: Int J Mol Sci. 2022 Jan 17;23(2):995. doi: 10.3390/ijms23020995 (PMC8780304; doi:10.3390/ijms23020995)
Supplement: Supplementary file 1 [file ijms-23-00995-s001.zip › ijms-1527933-supplementary.pdf]

TCD			Blueprint			
ABCA4	IMPDH1	RP1	ABCA4	EMC1	MVK	SLC24A1
ABCC6	IMPG1	RP1L1	ABCC6	**ESPN**	MYO7A	SLC25A46
ABHD12	IMPG2	RP2	**ABCD1**	EXOSC2	**NAGLU**	**SLC45A2**
**ACBD5**	INPP5E	**RP9**	ABHD12	EYS	NDP	SLC7A14
ADAM9	INVS	RPE65	**ACO2**	FAM161A	NEK2#	SNRNP200
ADAMTS18	IQCB1	RPGR	ADAM9	**FDXR**	NMNAT1#	SPATA7
**ADGRA3**	**ITM2B**	RPGRIP1	ADAMTS18	FLVCR1	NPHP1	SPP2
ADGRV1	JAG1	RPGRIP1L	ADGRV1	**FRMD7**	NPHP3	SRD5A3
ADIPOR1	KCNJ13	RS1	ADIPOR1	FZD4	NPHP4	**TCTN1#**
AGBL5	KCNV2	RTN4IP1	AGBL5	GNAT1	NR2E3	**TCTN2**
AHI1	KIAA1549	SAG	AHI1	GNAT2	NR2F1	**TCTN3**
AIPL1	KIF11	SDCCAG8	AIPL1	GNB3	NRL	TEAD1
ALMS1	KIZ	SEMA4A	ALMS1	GNPTG	NYX	TIMM8A
ARL2BP	KLHL7	SLC24A1	**AMACR**	**GPR143**	OAT	TIMP3
ARL3	LAMA1	SLC25A46	**ARHGEF18**	GPR179	**OCA2**	**TMEM107**
ARL6	LCA5	SLC7A14	**ARL13B**	GRK1	OFD1	TMEM126A
**ASRGL1**	LRAT	SNRNP200	ARL2BP	GRM6	OPA1	**TMEM138**
ATF6	LRIT3	SPATA7	ARL3	GUCA1A	OPA3	TMEM216
**ATXN7**	LRP5	SPP2	ARL6	GUCY2D	OPN1SW	**TMEM231**
BBIP1	LZTFL1	TEAD1	**ARMC9**	HARS	OTX2	TMEM237
BBS1	MAK	TIMM8A	**ARR3**	HGSNAT	**P3H2**	**TMEM67**
BBS10	**MAPKAPK3**	TIMP3	**ARSG**	HK1#	PANK2	TOPORS
BBS12	MERTK	TMEM126A	ATF6	HMX1	PAX2	**TPP1**
BBS2	MFN2	TMEM216	**ATOH7**	**IDH3A**	PCDH15	**TRAF3IP1**
BBS4	MFRP	TMEM237	**B9D1**	IDH3B	PCYT1A	TREX1
BBS5	MFSD8	TOPORS	**B9D2**	IFT140	PDE6A	TRIM32
BBS7	**MIR204**	TREX1	BBIP1	IFT172	PDE6B	TRPM1
BBS9	MKKS	TRIM32	BBS1	IFT27	PDE6C	TSPAN12
BEST1	MKS1	**TRNT1**	BBS10	**IFT81#**	**PDE6D**	**TTC21B**
**C12orf65**	MTTP	TRPM1	BBS12	IMPDH1	PDE6G	TTC8
C1QTNF5	MVK	TSPAN12	BBS2	IMPG1	PDE6H	TTLL5
C21orf2	MYO7A	TTC8	BBS4	IMPG2	**PDSS1#**	TTPA
C2orf71	**NBAS**	TTLL5	BBS5	INPP5E	**PDSS2**	TUB
C8orf37	NDP	TTPA	BBS7	INVS	PDZD7#	**TUBB4B**
CA4	NEK2	TUB	BBS9	IQCB1	PEX1	TUBGCP4
CABP4	**NEUROD1**	TUBGCP4	BEST1	**ISPD**	**PEX10**	TUBGCP6
CACNA1F	NMNAT1	TUBGCP6	C1QTNF5	JAG1	**PEX11B**	TULP1
CACNA2D4	NPHP1	TULP1	C21ORF2	KCNJ13	**PEX12**	**TYR**
CAPN5	NPHP3	**UNC119**	C2ORF71	KCNV2	**PEX13**	**TYRP1**
CC2D2A	NPHP4	USH1C	**C5ORF42**	**KIAA0556**	**PEX14**	USH1C
CDH23	NR2E3	USH1G	C8ORF37	**KIAA0586#**	**PEX16**	USH1G
CDH3	NR2F1	USH2A	CA4	**KIAA0753**	**PEX19**	USH2A
CDHR1	NRL	VCAN	CABP4	KIAA1549	PEX2	VCAN
CEP164	NYX	WDPCP	CACNA1F	KIF11	**PEX26**	**VPS13B**
CEP250	OAT	WDR19	CACNA2D4	**KIF7**	**PEX3**	WDPCP
CEP290	OFD1	WFS1	CAPN5	KIZ	**PEX5**	WDR19
CERKL	OPA1	**WHRN**	CC2D2A#	KLHL7	**PEX6**	WFS1
**CFH**	OPA3	ZNF408	CDH23	LAMA1	PEX7	**YME1L1**
CHM	**OPN1LW**	ZNF423	CDH3	LCA5	PHYH	ZNF408

CIB2	**OPN1MW**	ZNF513	CDHR1	LRAT	**PISD**	ZNF423
CLN3	OPN1SW		**CEP104**	LRIT3	PITPNM3	ZNF513
CLRN1	OTX2		**CEP120**	**LRP2**	PLA2G5	
**CLUAP1**	PANK2		CEP164	LRP5	PLK4	
CNGA1	PAX2		**CEP19**	LZTFL1	PNPLA6	
CNGA3	PCDH15		CEP250	MAK	POC1B	
CNGB1	PCYT1A		CEP290	MERTK	POMGNT1	
CNGB3	PDE6A		**CEP41**	MFN2	**PPT1**	
CNNM4	PDE6B		**CEP78**	MFRP	PRCD	
COL11A1	PDE6C		**CEP83**	MFSD8	PRDM13	
COL2A1	PDE6G		CERKL	MKKS	PROM1	
COL9A1	PDE6H		CHM#	MKS1	PRPF3	
CRB1	PDZD7		CIB2	**MMACHC**	PRPF31	
CRX	PEX1		**CISD2**	**MT-ATP6**	PRPF4	
CSPP1	PEX2		CLN3	**MT-ATP8**	PRPF6	
CTNNA1	PEX7		**CLN5**	**MT-CO1**	PRPF8	
CYP4V2	**PGK1**		**CLN6**	**MT-CO2**	PRPH2	
DHDDS	PHYH		**CLN8**	**MT-CO3**	PRPS1	
DHX38	PITPNM3		CLRN1	**MT-CYB**	RAB28	
**DMD**	PLA2G5		CNGA1#	**MT-ND1**	RAX2	
DRAM2	PLK4		CNGA3	**MT-ND2**	RBP3	
DTHD1	PNPLA6		CNGB1	**MT-ND3**	RBP4	
EFEMP1	POC1B		CNGB3	**MT-ND4**	RCBTB1	
ELOVL4	POMGNT1		CNNM4	**MT-ND4L**	RD3	
EMC1	PRCD		COL11A1	**MT-ND5**	RDH11	
EXOSC2	PRDM13		**COL11A2**	**MT-ND6**	RDH12	
EYS	PROM1		**COL18A1**	**MT-RNR1**	RDH5	
FAM161A	PRPF3		COL2A1	**MT-RNR2**	**REEP6**	
FLVCR1	PRPF31		COL9A1	**MT-TA**	RGR	
**FSCN2**	PRPF4		**COL9A2**	**MT-TC**	RGS9	
FZD4	PRPF6		**COL9A3**	**MT-TD**	RGS9BP	
**GDF6**	PRPF8		**COQ2**	**MT-TE**	RHO	
GNAT1	PRPH2		**CPE**	**MT-TF**	RIMS1	
GNAT2	PRPS1		CRB1	**MT-TG**	RLBP1	
GNB3	RAB28		CRX	**MT-TH**	ROM1	
GNPTG	RAX2		CSPP1	**MT-TI**	RP1	
GPR179	**RB1**		**CTC1**	**MT-TK**	RP1L1	
GRK1	RBP3		CTNNA1	**MT-TL1**	RP2	
GRM6	RBP4		**CTNNB1**	**MT-TL2**	RPE65	
GUCA1A	RCBTB1		**CTSD**	**MT-TM**	RPGR	
**GUCA1B**	RD3		**CWC27**	**MT-TN**	RPGRIP1	
GUCY2D	RDH11		CYP4V2	**MT-TP**	RPGRIP1L#	
HARS	RDH12		**DFNB31**	**MT-TQ**	RS1	
HGSNAT	RDH5		DHDDS	**MT-TR**	RTN4IP1	
HK1	RGR		DHX38	**MT-TS1**	SAG	
**HMCN1**	RGS9		**DNAJC5**	**MT-TS2**	**SAMD11**	
HMX1	RGS9BP		DRAM2	**MT-TT**	**SCAPER**	
IDH3B	RHO		DTHD1	**MT-TV**	**SCLT1#**	
IFT140	RIMS1		**DYNC2H1**	**MT-TW**	SDCCAG8	
IFT172	RLBP1		EFEMP1	**MT-TY**	SEMA4A	

IFT27	ROM1		ELOVL4	MTTP	**SGSH**	

Supplementary Table S1. List of the genes included on IRD panels for the research laboratory (TCD, 250 genes) and the commercial lab (Blueprint Genetics, 352 genes). Genes in **bold** text in each respective panel are not found in the other panel.
